# Emergence of *Acinetobacter soli* harboring three carbapenemase-encoding genes (*bla*_NDM-1_, *bla*_IMP-14_, and *bla*_OXA-58_) on a single plasmid in an ICU patient

**DOI:** 10.1128/spectrum.01130-26

**Published:** 2026-06-23

**Authors:** Qiao Li, Nanqi Zhang, Hao Guo, Juan Xu, Andrey Bakunovich, Wenping Lin, Fang He

**Affiliations:** 1 Laboratory Medicine Center, Department of Clinical Laboratory, Zhejiang Provincial People's Hospital (Affiliated People's Hospital), Hangzhou Medical College 117839, Hangzhou, Zhejiang, China; 2 School of Basic Medical Sciences and Forensic Medicine, Hangzhou Medical College 117839, Hangzhou, Zhejiang, China; 3 Department of Environmental Chemistry and Biochemistry, International Sakharov Environmental Institute of Belarusian State University 118899, Minsk, Belarus; 4 Centers for Disease Control and Prevention of Ningbo, Ningbo, Zhejiang, China; University of Technology Sydney, Sydney, Australia

**Keywords:** *Acinetobacter soli*, carbapenemase, *bla*_OXA-58_, *bla*_NDM-1_, *bla*_IMP-14_

## Abstract

*Acinetobacter soli* is an environmentally adaptable species increasingly recognized as an emerging pathogen in hospital settings, particularly in intensive care units (ICUs). In this study, we report the first *A. soli* isolate from an ICU patient that co-harbors three carbapenemase-encoding genes (*bla*_NDM-1_, *bla*_IMP-14_, and *bla*_OXA-58_) on a single plasmid. Whole-genome sequencing revealed that multidrug resistance in this strain is mediated by a 294,790 bp plasmid, pSLAB-A, carrying 16 antimicrobial resistance genes, including all three carbapenemases. Comparative plasmid analysis showed a highly conserved backbone but identified a unique ~40 kb multidrug-resistance region containing *bla*_NDM-1_, *bla*_IMP-14_, and eight additional resistance genes. Genetic context analysis indicated that insertion sequences (IS*Aba125* and IS*Aba3*) and class 1 integrons contribute to the mobilization and accumulation of carbapenemase-encoding genes. Plasmid stability assays demonstrated that pSLAB-A remained stably maintained for more than 90 generations without antibiotic selection. A global survey of the NCBI database identified 15 *A. soli* strains carrying carbapenemase-encoding genes, most of which were isolated from China, with clinical specimens representing the predominant source. Seven carbapenemase-encoding genes were detected, with *bla_NDM-1_* being the most prevalent. Among eight isolates with complete genomes, all carried carbapenemase-encoding genes on plasmids. Phylogenetic analysis revealed regional dissemination of a clonal lineage across hospitals in Zhejiang Province and sustained nosocomial transmission within a hospital in Taiwan. These findings suggest that the spread of carbapenem resistance in *A. soli* is largely driven by multidrug-resistance plasmids, facilitating clonal expansion in hospital environments and posing a growing challenge for antimicrobial therapy and infection control in ICUs.

**IMPORTANCE:**

Carbapenem-resistant *A. soli* is an emerging clinical concern, capable of causing severe invasive infections, including bacteremia, in intensive care unit settings, and its emergence poses substantial challenges to antimicrobial therapy. In this study, we demonstrate that carbapenem resistance in *A. soli* is predominantly mediated by the acquisition of multidrug-resistance plasmids carrying carbapenemase-encoding genes. Owing to its strong environmental persistence, *A. soli* can readily undergo nosocomial clonal dissemination once carbapenem resistance is acquired. Moreover, the spread of multidrug plasmids co-harboring multiple carbapenemase-encoding genes may accelerate the evolutionary trajectory of resistance in *A. soli*, further exacerbating the threat to clinical management. Given its demonstrated capacity to cause hospital-associated infections and to rapidly acquire multidrug resistance, *A. soli* warrants heightened vigilance from both clinical and public health perspectives.

## INTRODUCTION

The genus *Acinetobacter* comprises pleomorphic gram-negative coccobacilli and is a well-recognized cause of healthcare-associated infections, particularly in intensive care units (ICUs). Infections caused by *Acinetobacter* spp. include ventilator-associated pneumonia, bloodstream infections, and urinary tract infections, imposing a substantial burden in hospital settings (1–4). *Acinetobacter soli*, an environmentally adaptable member of this genus, was first identified in 2008 from forest soil in South Korea (5). Since then, its clinical relevance has become increasingly evident. In 2011, an outbreak of clonally related *A. soli* isolates causing bloodstream infections in a neonatal ICU (involving five neonates) was reported (6). The first carbapenem-resistant *A. soli* isolate in China was described in 2014, recovered from a patient with ICU-associated bloodstream infection (7). More recently, in 2023, carbapenem-resistant *A. soli* strains isolated from patients with bacteremia were reported in Japan (8). These findings indicate that *A. soli* is capable of causing severe infections in ICU settings and may represent an emerging clinical threat.

According to 2024 data from the China Antimicrobial Resistance Surveillance System (CHINET), resistance rates of *Acinetobacter* spp. to the carbapenems meropenem and imipenem reached 64.7% and 64.5%, respectively (9). The global emergence of carbapenem-resistant *Acinetobacter* has therefore severely restricted available therapeutic options (10, 11). Carbapenem resistance in *Acinetobacter* spp. is primarily mediated by the production of carbapenemases that hydrolyse carbapenems (12, 13). Based on the Ambler classification, β-lactamases are divided into four molecular classes (A–D), of which classes A, C, and D are serine β-lactamases, whereas class B comprises metallo-β-lactamases; carbapenemases are mainly distributed among classes A, B, and D (14, 15).

In this study, we identified a carbapenem-resistant *A. soli* strain SLAB, isolated from a male patient with a urinary tract infection, which co-harbored three carbapenemase-encoding genes, *bla*_NDM-1_, *bla*_IMP-14_, and *bla*_OXA-58_. Whole-genome sequencing and bioinformatic analyses were conducted to characterize its genomic features, with particular emphasis on the genetic contexts, chromosomal or plasmid localization, and potential transmission mechanisms of these carbapenemase-encoding genes. Furthermore, we searched the NCBI database and performed a global epidemiological analysis of all available carbapenemase-producing *A. soli* strains.

## MATERIALS AND METHODS

### Patient and isolate

The *A. soli* strain SLAB was isolated in 2019 from a urine sample of a male patient admitted to the intensive care unit of a tertiary hospital in Hangzhou, China. The patient was initially admitted to the Department of Hematology with a diagnosis of myelodysplastic syndrome and underwent hematopoietic stem cell transplantation. On hospital day 117, he developed progressive dyspnea and worsening pulmonary inflammation, necessitating endotracheal intubation and transfer to the ICU. *A. soli* was isolated from the urine sample 2 days after ICU admission. The isolate exhibited heavy growth with no mixed flora, indicating a pure culture. Notably, the patient did not have an indwelling urinary catheter, suggesting that the infection was unlikely to be catheter associated. Species identification was initially performed using the VITEK MS system (bioMérieux, France) and subsequently confirmed by whole-genome sequencing, with species assignment validated through comparison with reference genomes using the JSpeciesWS platform.

### Antimicrobial susceptibility test

Antimicrobial susceptibility testing of strain SLAB was performed using the VITEK 2 system with the AST-GN13 card and the standard broth microdilution method, in accordance with the Clinical and Laboratory Standards Institute (CLSI) guidelines (CLSI M100, 34th edition). The antimicrobial agents tested included ampicillin/sulbactam, ceftriaxone, ceftazidime, cefepime, imipenem, meropenem, tobramycin, gentamicin, levofloxacin, ciprofloxacin, trimethoprim–sulfamethoxazole, and tigecycline. Susceptibility interpretations were based on CLSI-defined breakpoints, except for tigecycline, for which the U.S. Food and Drug Administration criteria were applied.

### Whole-genome sequencing

Genomic DNA was extracted using the QIAamp DNA Mini Kit (Qiagen, USA). Whole-genome sequencing was performed using the Illumina NovaSeq 6000 platform (Illumina Inc., San Diego, CA, USA) and the Oxford Nanopore MinION sequencer (Oxford Nanopore Technologies, Oxford, UK). Illumina sequencing was conducted in 150 bp paired-end mode, achieving an average coverage depth of ≥100×, while Nanopore sequencing yielded an average coverage depth of ≥50×. Hybrid *de novo* assembly was carried out using Unicycler v0.5.0, followed by genome annotation with the NCBI Prokaryotic Genome Annotation Pipeline (PGAP).

### Bioinformatics analysis

Antibiotic resistance genes were identified using ABRicate v0.8.10 with the ResFinder database, applying thresholds of ≥90% identity and ≥90% coverage for resistance gene identification (16). Insertion sequences (ISs) on plasmids were predicted using ISfinder (17). Plasmid conjugative transfer ability was predicted using VRprofile2 (18). The genetic contexts of *bla*_NDM-1_ among different plasmids were compared using EasyFig v2.2.3 (19). Plasmids showing similarity to the carbapenemase gene-carrying plasmid identified in strain SLAB (pSLAB-A) were retrieved from the NCBI database. Circular comparative analyses of carbapenem resistance gene-bearing plasmids were performed using the BLAST Ring Image Generator v0.95, with results visualized as concentric ring maps (20). Plasmid replicon typing was performed using the Acinetobacter Plasmid Typing (APT) database version 3.0.0. Replication initiation (rep) gene sequences were clustered at a 95% nucleotide identity threshold, and representative Rep/rep sequences from the database were used for type assignment (21, 22).

### Phylogenetic analysis

To investigate the global epidemiology of carbapenem-resistant *Acinetobacter soli*, we searched the NCBI GenBank database using the keyword “*Acinetobacter soli*” (accessed October 2025) and retrieved all publicly available genome assemblies. Only entries annotated as “genome assembly” with downloadable FASTA files were included. Both complete and draft genomes were retained without exclusion based on assembly level to maximize data set coverage. A total of 84 *A. soli* genomes were identified worldwide, among which 15 carried carbapenemase-encoding genes. These 15 isolates were subjected to phylogenetic analysis using Snippy v4.6.0 for core-genome single-nucleotide polymorphism (SNP) calling and FastTree v2.1.11 for tree construction. The resulting phylogenetic tree was visualized using iTOL v7 (https://itol.embl.de).

### Conjugation experiments

The transferability of plasmid pSLAB-A was evaluated using a filter-mating conjugation assay, with rifampicin-resistant *A. baumannii* ATCC 17978 serving as the recipient strain (23). Transconjugants were selected on Mueller-Hinton (MH) agar plates supplemented with rifampicin (100 μg/mL) and meropenem (4 μg/mL). The identity of the transconjugants was further confirmed by PCR amplification followed by Sanger sequencing.

### Stability of the carbapenemase gene-carrying plasmid

The stability of the carbapenemase gene-carrying plasmid in *A. soli* was evaluated by serial passaging in the absence of antibiotic pressure. Briefly, a single colony of *A. soli* SLAB was inoculated into 3 mL of LB broth and incubated at 37°C with shaking for 24 h. Each day, 3 μL of the overnight culture was transferred into fresh LB broth at a 1:1,000 dilution. Under this dilution scheme, the culture increased from approximately 10^6^–10^9^ CFU/mL per day, corresponding to ~10 bacterial generations daily. This procedure was repeated for 9 consecutive days (approximately 90 generations). Every 3 days, cultures were serially diluted with sterile phosphate-buffered saline and plated onto MH agar without antibiotics, followed by overnight incubation at 37°C. Forty colonies were randomly selected from each time point, and the presence of *bla*_NDM-1_, *bla*_IMP-14_, and *bla*_OXA-58_ was determined by PCR. The proportion of colonies harboring all three genes among the 40 screened colonies was calculated to assess plasmid stability (24).

## RESULTS AND DISCUSSION

The minimum inhibitory concentrations of antimicrobial agents against *A. soli* strain SLAB are summarized in Table S1. Antimicrobial susceptibility testing showed that SLAB was resistant to ampicillin/sulbactam, ceftriaxone, ceftazidime, cefepime, imipenem, and meropenem; exhibited intermediate susceptibility to tobramycin and gentamicin; and remained susceptible to ciprofloxacin, levofloxacin, trimethoprim–sulfamethoxazole, and tigecycline.

The assembled genome of strain SLAB comprised seven contigs with a total length of 3,876,811 bp, including a circular chromosome of 3,459,198 bp and six circular plasmids of 294,790 bp, 97,614 bp, 12,013 bp, 5,480 bp, 4,868 bp, and 2,848 bp, respectively. PGAP annotation identified 21 rRNA genes and 76 tRNA genes. In addition, 17 antimicrobial resistance genes spanning seven antimicrobial classes were detected (Table 1), including β-lactamase genes (*bla*_NDM-1_, *bla*_OXA-58_, and *bla*_IMP-14_), macrolide resistance genes (*msr*(*E*) and *mph(E*)), aminoglycoside resistance genes (*aacA34*, *strB*, *strA*, *aadA2*, *aphA5a*, *aadB*, and *aac*(6')-Ib4), phenicol resistance genes (*floR* and *cmlA1*), the sulfonamide resistance gene *sul1*, the tetracycline resistance gene *tet*(39), and the quinolone resistance gene *qnrVC6*.

**TABLE 1:** Antimicrobial resistance genes identified in *A. soli* SLAB

Antimicrobial resistance gene	Contig	Identity (%)	Position in contig	Antimicrobial resistance category	Accession no. of reference gene
*bla*_OXA-58_	pSLAB-A	100	32416–33258	Beta-lactam	NG_049798.1
*bla*_NDM-1_	pSLAB-A	100	104919–105731	Beta-lactam	NG_049326.1
*bla*_IMP-14_	pSLAB-A	99.86	87925–88665	Beta-lactam	NG_049177.1
*aacA34*	pSLAB-A	100	87339–87779	Aminoglycoside	NG_047319.1
*strB*	pSLAB-A	97.61	90092–90928	Aminoglycoside	NG_047464.1
*strA*	pSLAB-A	97.7	90928–91753	Aminoglycoside	NG_056002.2
*aadA2*	pSLAB-A	100	91761–92552	Aminoglycoside	NG_051846.1
*aadB*	pSLAB-A	100	94159–94692	Aminoglycoside	NG_047387.1
*aphA5a*	pSLAB-A	100	114300–115079	Aminoglycoside	NG_047448.1
*aac*(6')*-Ib4*	pSLAB-A	100	133612–134166	Aminoglycoside	NG_051844.1
*floR*	pSLAB-A	99.92	10722–11936	Phenicol	NG_051844.1
*cmlA1*	pSLAB-A	99.6	92645–93904	Phenicol	NG_047647.1
*msr(E*)	pSLAB-A	99.8	17445–18923	Macrolide	NG_048007.1
*mph(E*)	pSLAB-A	100	18979–19863	Macrolide	NG_064660.1
*sul1*	pSLAB-A	100	86041–86880	Sulfonamide	NG_048082.1
*qnrVC6*	pSLAB-A	100	134330–134986	Quinolone	NG_050555.1
*tet*(39)	pSLAB-D	100	8255–9442	Tetracycline	NG_048137.1

**Fig 1:**
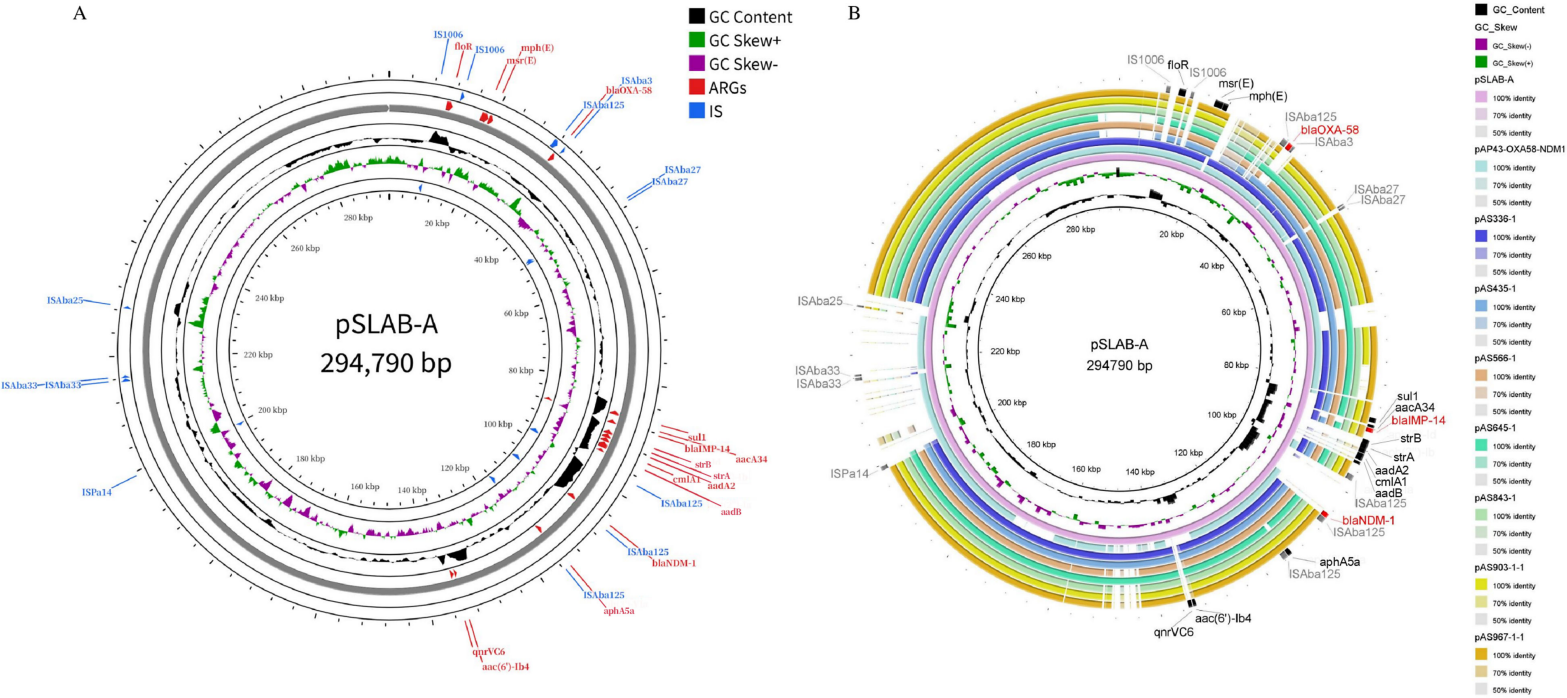
(**A**) Circular map of plasmid pSLAB-A from strain SLAB. Antimicrobial resistance genes are shown in red, and insertion sequences are indicated in blue. (**B**) Circular comparison of plasmid pSLAB-A with closely related plasmids retrieved from the NCBI database: pAS336-1 (*A. soli*, CP119218.1, 272,687 bp), pAS435-1 (*A. soli*, CP119229.1, 296,393 bp), pAS645-1 (*A. soli*, CP119239.1, 299,645 bp), pAS967-1-1 (*A. soli*, CP119216.1, 324,273 bp), pAS566-1 (*A. soli*, CP119251.1, 325,311 bp), pAS843-1 (*A. soli*, CP119204.1, 324,240 bp), pAS903-1-1 (*A. soli*, CP119208.1, 317,471 bp), and pAP43-OXA58-NDM1 (*A. pittii*, CP043053.1, 268,263 bp).

Among the 17 antimicrobial resistance genes identified in *A. soli* SLAB, all except *tet*(39) are located on plasmid pSLAB-A (294,790 bp), including the three carbapenemase-encoding genes *bla*_OXA-58_, *bla*_NDM-1_, and *bla*_IMP-14_. Plasmid pSLAB-A is 294,790 bp in length with a GC content of 39% and harbors 300 predicted coding sequences. Its circular map is shown in Fig. 1A. Analysis using the PlasmidFinder database failed to identify any known plasmid replicon. Gene annotation revealed that most antimicrobial resistance genes on pSLAB-A are organized into a large resistance cluster. Within this cluster, *bla*_NDM-1_ and *bla*_IMP-14_ are concentrated within an approximately 40 kb region (from ~80 kb to ~120 kb). This region also contains multiple additional resistance genes, including *sul1*, *aacA34*, *strB*, *strA*, *aadA2*, *cmlA1*, *aadB*, and *aphA5a*. In contrast, *bla*_OXA-58_ is located in a relatively discrete region of the plasmid. Conjugation assays showed that plasmid pSLAB-A was not transferable to the recipient strain. Screening for conjugation-associated genes, including components of the type IV secretion system, identified none, suggesting that the absence of essential transfer-related elements likely underlies the lack of transconjugants. Notably, serial passage assays demonstrated that pSLAB-A remained highly stable, with retention rates of 100% at day 3, 97% at day 6, and 91% at day 9.

**Fig 2:**
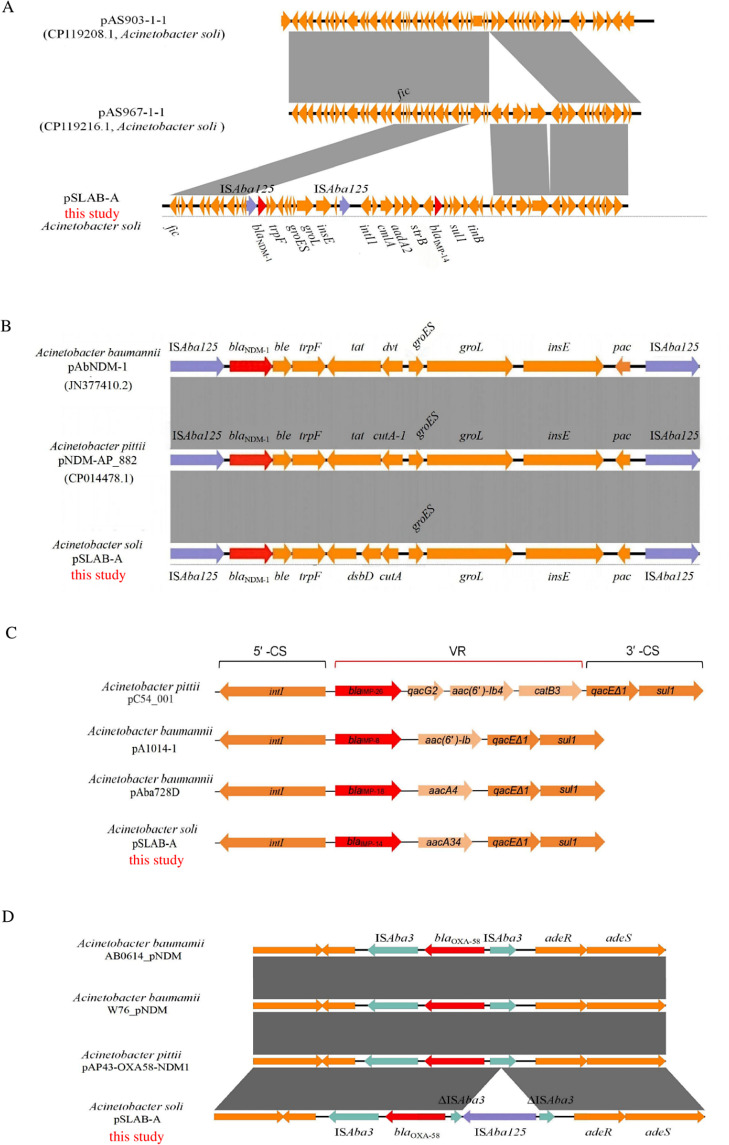
(**A**) Comparative analysis of plasmids pAS903-1-1, pAS967-1-1, and pSLAB-A. Antimicrobial resistance genes are shown in red, and insertion sequences are indicated in purple. (**B**) Comparative analysis of the *bla*_NDM-1_ genetic context. Shaded regions represent sequence homology across corresponding loci on different plasmids. Arrows indicate gene length and orientation and are labeled with gene names; *bla*_NDM-1_ is highlighted in red, and IS*Aba125* is shown in purple. (**C**) Schematic representation of distinct class 1 integrons carrying the *bla*_IMP_ gene in *Acinetobacter* spp. Arrows indicate the direction of gene transcription. The 5′ conserved segment (5′-CS) and 3′ conserved segment (3′-CS) are highlighted in orange, while the variable region is shown in light orange. The *bla*_IMP_ gene is displayed in red. (**D**) Structural comparison of *bla*_OXA-58_–harboring regions among plasmids pAP43-OXA58-NDM1, W76_pNDM, AB0614_pNDM, and pSLAB-A. Green arrows indicate IS*Aba3*, the *bla*_OXA-58_ gene is shown in red, and purple arrows represent IS*Aba125*.

We further analyzed the genetic environments surrounding the three carbapenemase-encoding genes. The *bla*_NDM-1_ gene was found to be located within a conserved 10,038 bp segment flanked by two IS*Aba125* insertion sequences, forming the composite transposon Tn*125* with the structure “IS*Aba125 - bla*_NDM-1_ - *ble*_MBL_ - *trpF - dsbD - cutA - groES - groL - insE* - IS*Aba125*” (Fig. 2A and B). This genetic structure showed complete sequence conservation (100% coverage and 100% identity) with corresponding regions identified in *Acinetobacter* spp. genomes with accession numbers JN377410.2 and CP014478.1 (Fig. 2B). Comparative analysis with related plasmids retrieved from the NCBI database, including pAS903-1-1 (accession numbers CP119208.1) and pAS967-1-1 (accession number CP119216.1), suggested that the *bla*_NDM-1_-containing region in plasmid pSLAB-A was likely acquired through IS*Aba125*-mediated transposition (Fig. 2A).

The *bla*_IMP-14_ gene is embedded within a canonical class 1 integron comprising a 5′ conserved segment (*intI1*), a variable gene cassette array containing *bla*_IMP-14_ and additional antimicrobial resistance genes, and a 3′ conserved segment (*qacEΔ1–sul1*; Fig. 2C). This integron architecture represents a highly efficient genetic platform for the horizontal dissemination of *bla*_IMP-14_ among *Acinetobacter* lineages (25).

The *bla*_OXA-58_ gene is classically bracketed by two IS*Aba3* elements. In plasmid pSLAB-A, however, the upstream IS*Aba3* is truncated by insertion of IS*Aba125*, whereas the downstream IS*Aba3* remains intact (Fig. 2D). This rearrangement is functionally significant, as IS*Aba125* provides a strong outward-facing promoter that drives overexpression of *bla*_OXA-58_, thereby markedly enhancing carbapenem resistance, as previously demonstrated (26).

Using pSLAB-A as the reference, BLASTn searches were performed against the NCBI database with thresholds of >99% nucleotide identity and ≥70% query coverage, yielding eight closely related plasmids (Table S2): pAS336-1 (CP119218.1), pAS435-1 (CP119229.1), pAS645-1 (CP119239.1), pAS967-1-1 (CP119216.1), pAS566-1 (CP119251.1), pAS843-1 (CP119204.1), pAS903-1-1 (CP119208.1), and pAP43-OXA58-NDM1 (CP043053.1). Among these, pAP43-OXA58-NDM1 originated from *Acinetobacter pittii*, whereas all remaining plasmids were recovered from *A. soli*. The plasmid showing the highest similarity to pSLAB-A was pAS336-1 (99.79% identity and 82% coverage), which was isolated from *A. soli* strain AS336 recovered from a bloodstream infection in Taiwan, China, in 2001. A concentric circular comparison of pSLAB-A with these homologous plasmids is shown in Fig. 1B. Notably, eight plasmids in our data set could not be assigned to any APT Rep type (Table S4), consistent with previous reports that plasmids lacking known Rep sequences are associated with the dissemination of important antimicrobial resistance genes (22). This analysis revealed a highly conserved plasmid backbone shared across all plasmids, with a notable exception in pSLAB-A: a unique resistance-enriched region of approximately 40 kb (from 80 kb to 120 kb) that harbors *bla*_NDM-1_, *bla*_IMP-14_, and eight additional antimicrobial resistance genes.

**Fig 3:**
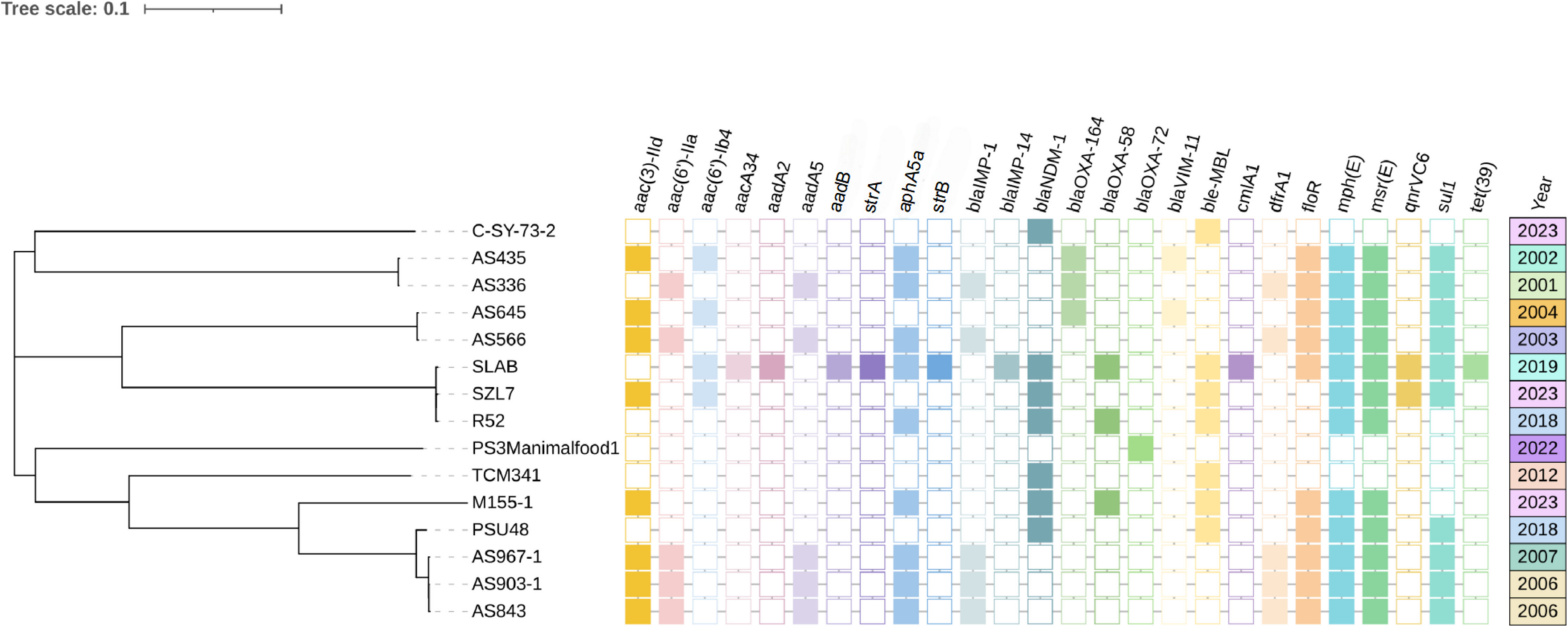
Phylogenetic analysis of strain SLAB and 14 additional *A. soli* strains carrying carbapenemase-encoding genes retrieved from the NCBI database. Colored cells indicate the presence of specific antimicrobial resistance genes, whereas blank cells denote their absence. Resistance genes are grouped and annotated at the top of the figure. Sampling dates are color coded. Branch labels correspond to strain names, and the scale bar represents SNP divergence among strains.

A global search of the NCBI database identified 15 *A. soli* strains carrying carbapenemase-encoding genes (Table S3). Except for one isolate from Thailand, all others originated from China, including Taiwan (*n* = 7), Zhejiang (*n* = 6), and Guangdong (*n* = 1). Across these isolates, seven distinct carbapenemases were detected: *bla*_OXA-164_, *bla*_OXA-58_, *bla*_OXA-72_, *bla*_NDM-1_, *bla*_IMP-1_, *bla*_IMP-14_, and *bla*_VIM-11_ (Fig. 3). *bla*_NDM-1_ was the most prevalent (46.7%, 7/15), followed by *bla*_IMP-1_ (33.3%, 5/15), *bla*_OXA-164_ (20.0%, 3/15), *bla*_OXA-58_ (20.0%, 3/15), *bla*_VIM-11_ (13.3%, 2/15), *bla*_OXA-72_ (6.7%, 1/15), and *bla*_IMP-14_ (6.7%, 1/15). Nine isolates carried a single carbapenemase gene, five carried two, whereas only strain SLAB co-harbored three carbapenemases. Among the 15 isolates, one originated from livestock, one from the environment, and the remaining 13 from clinical specimens, predominantly bloodstream infections (7/15, 46.7%). Complete genome sequences were available for eight isolates, all of which harbored their carbapenemase-encoding genes on plasmids. Phylogenetic analysis (Fig. 3) revealed that strain SLAB clustered with strains R52 and SZL7 within the same clonal lineage, differing by 59 and 62 SNPs, respectively (Fig. S1). Notably, R52 and SZL7 were isolated from clinical specimens collected in 2018 and 2023 from two independent hospitals in Zhejiang Province, indicating regional dissemination of this lineage. In addition, seven *A. soli* isolates were recovered from a single hospital in Taiwan over a 6-year period (2001–2007). Among these, three isolates (AS843, AS903-1, and AS967-1), collected between 2006 and 2007, formed a tight clonal cluster with ≤35 SNP differences, strongly suggesting nosocomial clonal transmission within the hospital.

Collectively, this study provides the first report of *A. soli* co-harboring three carbapenemase-encoding genes (*bla*_NDM-1_, *bla*_OXA-58_, and *bla*_IMP-14_) on a single plasmid in an ICU setting. The mobilization and accumulation of these carbapenemase-encoding genes are mediated by IS*Aba125*, IS*Aba3*, and class 1 integrons, underscoring their pivotal roles in inter- and intraplasmid dissemination. Global genomic evidence further indicates that the emergence and spread of carbapenem resistance in *A. soli* is primarily driven by the acquisition of multidrug-resistance plasmids, which can fuel clonal expansion within hospital environments. These data underscore that plasmids play a central role in disseminating and accumulating antimicrobial resistance within this species. The propagation of such high-risk plasmids in *A. soli* poses a serious threat to antimicrobial therapy and represents an escalating challenge for infection control in intensive care units.

## Data Availability

The whole-genome results of strain SLAB have been deposited in DDBJ/EMBL/GenBank under accession numbers JBRAZX010000001–JBRAZX010000007.

## SUPPLEMENTAL MATERIAL

Supplemental figure and tables spectrum.01130-26-s0001.docx Fig. S1 and Tables S1 to S4.

ASM does not own the copyrights to Supplemental Material that may be linked to, or accessed through, an article. The authors have granted ASM a non-exclusive, world-wide license to publish the Supplemental Material files. Please contact the corresponding author directly for reuse.
