# Supplementary material for: Emergence of Acinetobacter soli harboring three carbapenemase-encoding genes (blaNDM-1, blaIMP-14, and blaOXA-58) on a single plasmid in an ICU patient
Source: Microbiol Spectr. 2026 Jun 23;14(8):e01130-26. doi: 10.1128/spectrum.01130-26 (PMC13435676; doi:10.1128/spectrum.01130-26)
Supplement: Supplementary file 1 [file spectrum.01130-26-s0001.docx]

Table S1. The minimum inhibitory concentration (MICs) of the antibiotics tested in *A.soli* SLAB.

| Antibiotics | MIC(mg/L) | |
| --- | --- | --- |
|  | SLAB | Interpretive categories |
| Ampicillin/Sulbactam^a^ | ≥32 | R |
| Ceftriaxone^a^ | ≥64 | R |
| Ceftazidime^a^ | ≥64 | R |
| Cefepime^a^ | ≥64 | R |
| Imipenem^a^ | ≥16 | R |
| Meropenem^b^ | ≥64 | R |
| Tobramycin^a^ | 8 | I |
| Gentamicin^a^ | 8 | I |
| Levofloxacin^a^ | 1 | S |
| Ciprofloxacin^a^ | 1 | S |
| Trimethoprim/Sulfamethoxazole^a^ | ≥1/19 | S |
| Tigecycline^b^ | 1 | S |

^a^Tested by AST-GN13

^b^Tested by the standard broth microdilution method

Abbreviation: R, resistant; I, Intermediate; S, Susceptible.

Table S2. Information on eight plasmids with significant sequence homology to plasmid pSLAB-A from the NCBI database.

| **Plasmids** | **Strain** | **Size (bp)** | **G+C content (mol%)** | **Carbapenemase-encoding genes** | **Accession number** |
| --- | --- | --- | --- | --- | --- |
| pAS336-1 | AS336 | 272,687 | 39 | *bla*_OXA-164_, *bla*_IMP-1_ | CP119218.1 |
| pAS435-1 | AS435 | 296,393 | 40 | *bla*_OXA-164,_ *bla*_VIM-11_ | CP119229.1 |
| pAS645-1 | AS645 | 299,645 | 40 | *bla*_OXA-164_, *bla*_VIM-11_ | CP119239.1 |
| pAS967-1-1 | AS967-1 | 324,273 | 40 | *bla*_IMP-1_ | CP119216.1 |
| pAS566-1 | AS566 | 325,311 | 40 | *bla*_IMP-1_ | CP119251.1 |
| pAS843-1 | AS843 | 324,240 | 40 | *bla*_IMP-1_ | CP119204.1 |
| pAS903-1-1 | AS903-1 | 317,471 | 40 | *bla*_IMP-1_ | CP119208.1 |
| pAP43-OXA58-NDM1 | AP43 | 268,263 | 39 | *bla*_NDM-1_, *bla*_OXA-58_ | CP043053.1 |

Table S3. Information of 15 strains of *A. soli* retrieved from the NCBI database harboring carbapenemase genes.

| **Assembly Accession** | **Strain** | **Geographic location** | **Isolation type** | **Isolation Source** | **Carbapenemase-encoding genes** | **Collection Date** |
| --- | --- | --- | --- | --- | --- | --- |
| GCA_000633005.1 | TCM341 | China: Zhejiang | Sputum | clinical | *bla*_NDM-1_ | 2012 |
| GCA_021384325.1 | R52 | China: Zhejiang | - | clinical | *bla*_OXA-58_, *bla*_NDM-1_ | 2018 |
| GCA_029168355.1 | AS566 | China: Taiwan | blood | clinical | *bla*_IMP-1_ | 2003 |
| GCA_029168375.1 | AS843 | China: Taiwan | blood | clinical | *bla*_IMP-1_ | 2006 |
| GCA_029168415.1 | AS903-1 | China: Taiwan | blood | clinical | *bla*_IMP-1_ | 2006 |
| GCA_029168435.1 | AS967-1 | China: Taiwan | blood | clinical | *bla*_IMP-1_ | 2007 |
| GCA_029168495.1 | AS645 | China: Taiwan | blood | clinical | *bla*_OXA-164_, *bla*_VIM-11_ | 2004 |
| GCA_029168515.1 | AS336 | China: Taiwan | blood | clinical | *bla*_OXA-164_, *bla*_IMP-1_ | 2001 |
| GCA_029168535.1 | AS435 | China: Taiwan | blood | clinical | *bla*_OXA-164,_ *bla*_VIM-11_ | 2002 |
| GCA_030541015.1 | M155-1 | China: Zhejiang | - | clinical | *bla*_OXA-58_, *bla*_NDM-1_ | 2023 |
| GCA_031158155.1 | C-SY-73-2 | China: Zhejiang | sewage water | environmental | *bla*_NDM-1_ | 2023 |
| GCA_036940535.1 | PSU48 | Thailand | - | clinical | *bla*_NDM-1_ | 2018 |
| GCA_048282975.1 | PS3Manimalfood1 | China: Guangdong | Animal food | livestock | *bla*_OXA-72_ | 2022 |
| GCA_052805795.1 | SLAB | China: Zhejiang | urine | clinical | *bla*_OXA-58_, *bla*_NDM-1_, *bla*_IMP-14_ | 2019 |
| GCA_049195125.1 | SZL7 | China: Zhejiang | sputum | clinical | *bla*_NDM-1_ | 2023 |

Table S4. Characteristics and Rep family classification of the plasmids analyzed in this study.

| **Plasmids** | **Size (bp)** | **APT typing** | **Nucleotide identity** | **Rep family** |
| --- | --- | --- | --- | --- |
| pSLAB-A | 294,790 | / | No match | R3(PFO1051) |
| pAS336-1 | 272,687 | / | No match | R3(PFO1051) |
| pAS435-1 | 296,393 | / | No match | R3(PFO1051) |
| pAS645-1 | 299,645 | / | No match | R3(PFO1051) |
| pAS967-1-1 | 324,273 | / | No match | R3(PFO1051) |
| pAS566-1 | 325,311 | / | No match | R3(PFO1051) |
| pAS843-1 | 324,240 | / | No match | R3(PFO1051) |
| pAS903-1-1 | 317,471 | / | No match | R3(PFO1051) |
| pAP43-OXA58-NDM1 | 268,263 | / | No match | R3(PFO1051) |

**Figure S1.** The heatmap shows the number of SNPs differences between each isolate, with darker colours indicating greater differences.
